# Explanatory integration and integrated explanations in Darwinian medicine and evolutionary medicine

**DOI:** 10.1007/s11017-022-09594-z

**Published:** 2022-10-29

**Authors:** Nina Kranke

**Affiliations:** Westfälische Wilhelms-Universität Münster, Münster, Germany

**Keywords:** Darwinian medicine, Interdisciplinary integration, Evolutionary medicine, Explanatory integration, Old friends hypothesis, Antibiotic resistance, Experimental evolution

## Abstract

Recently, two research traditions that bring together evolutionary biology and medicine, that is to say, Darwinian medicine and evolutionary medicine, have been identified. In this paper, I analyse these two research traditions with respect to explanatory and interdisciplinary integration. My analysis shows that Darwinian medicine does not integrate medicine and evolutionary biology in any strong sense but does incorporate evolutionary concepts into medicine. I also show that backward-looking explanations in Darwinian medicine are not integrated proximate-and-ultimate explanations but functional explanations that include reference to evolutionary concepts. Nevertheless, explanations in Darwinian medicine have heuristic roles as they potentially contribute to conceptual change and tie pieces of knowledge from different fields of medical research together. I argue that Darwinian medicine is an “interfield” that fosters cross-disciplinary exchange between evolutionary biologists and medical researchers and practitioners based on division of labour and separation, rather than unity. Research in evolutionary medicine, on the other hand, happens at the intersection of evolutionary biology and medicine where the two disciplines are already integrated and is designed to produce entangled proximate-evolutionary explanations. My analysis thus adds another important aspect to the philosophical discussion on the distinction between Darwinian medicine and evolutionary medicine.

## Introduction

In the past three decades, calls for the integration of evolutionary biology and medicine have become increasingly pronounced. Darwinian medicine is one of the research traditions that aims at achieving this goal. Rephrasing Dobzhansky’s [1] famous dictum, proponents of Darwinian medicine even claim that “nothing in medicine makes sense except in the light of evolution” [2, p. 249; see also 3]. This conviction is based on the assumption that our bodies are shaped by evolutionary dynamics which has left them vulnerable to disease [4, p. 3]. Advocates of Darwinian medicine argue that an evolutionary approach offers a perspective that complements studies of proximate causes of health and disease [5, 6] because evolutionary biology studies fitness (i.e., reproductive success) of populations, while medicine is concerned with health (in the sense of overall wellbeing) of the individual [5, 7].

Based on Ernst Mayr [8] and Nikolaas Tinbergen`s [9] distinction of ultimate and proximate causes, proponents of Darwinian medicine argue that medicine answers “how” questions while evolutionary biology provides answers to the question of why we get sick: “Proximate explanations address how the body works and why some people get a disease and others don’t. Evolutionary explanations show why humans, in general, are susceptible to some diseases and not to others” [2, p. 6]. Advocates of Darwinian medicine want to bring about interdisciplinary integration of evolutionary biology and medicine by means of a synthesis of ultimate and proximate explanations to create more complete explanations and to contribute to a better understanding of human diseases. Unlike Darwinian medicine, evolutionary medicine does not have strong internal cohesion in terms of epistemology and methodology and some researchers who study the dynamics of infectious diseases and host-pathogen coevolution “are engaged in evolutionary medicine sometimes without knowing it” [10, p. 77]. Because of the rather loose association of researchers in evolutionary medicine there are no agreed-upon goals or approaches with respect to interdisciplinary or explanatory integration.

In what follows, I analyse Darwinian medicine and evolutionary medicine with respect to interdisciplinary and explanatory integration. As an exemplary explanation in Darwinian medicine, I discuss the so-called “old-friends hypothesis”. My analysis shows that Darwinian medicine does not produce integrated proximate-and-ultimate explanations, but functional explanations with reference to evolutionary concepts. Nevertheless, these explanations are useful tools that tie together results from different fields of medical research, and potentially contribute to conceptual change, opening up new avenues of research. I argue that Darwinian medicine can still play an important role in promoting cross-disciplinary exchange despite being based on separation and division of labour rather than blending or unification. Evolutionary medicine, on the other hand, generates entangled proximate-evolutionary explanations. The example of an experimental evolution study of antibiotic resistance shows that in evolutionary medicine, interdisciplinary integration precedes explanatory integration.

## Explanations in Darwinian medicine and evolutionary medicine

To date, explanations and explanatory practices in Darwinian medicine and evolutionary medicine have not received much philosophical scrutiny. In this section, I summarize the two main accounts by Pierre-Olivier Méthot [10] and Michael Cournoyea [14, 15] as they provide a basis for my analysis of explanatory and interdisciplinary integration within the two research traditions.

Méthot [10, p. 76] introduces the distinction between evolutionary medicine and Darwinian medicine and characterizes the two approaches as distinct “research traditions”. He argues that Darwinian medicine is a theoretically oriented approach, while researchers in evolutionary medicine are interested in finding practical solutions to medical problems by applying evolutionary reasoning or methods from evolutionary biology [10, p. 77]. Both approaches are similar in the sense that they apply evolutionary reasoning to explain, prevent and treat human diseases [11]. Darwinian medicine is a rather unified research tradition characterised by a set of theoretical and methodological commitments, for example, adaptationism as a heuristic principle in medicine and the assumption that humans are adapted to the environment of the Pleistocene savannah and maladapted to modern environments (mismatch hypothesis) [2, pp. 9, 21–25; 4, pp. 13–14; 6, p. 4305; 10, p. 78; 12; 13]. Evolutionary medicine, on the other hand, is not a cohesive scientific field, but rather “a collection of different research agendas” that apply evolutionary theory or methods from evolutionary biology [10, p. 77].

Méthot emphasises the different types of explanation generated by the two research traditions [10]. He argues that proponents of Darwinian medicine mostly generate backward-looking explanations, while practitioners of evolutionary medicine primarily produce forward-looking explanations. According to Méthot:

a forward looking explanation tries to predict the effects of ongoing evolutionary processes on human health and disease in contemporary environments (e.g., hospitals). In contrast, a backward looking explanation typically applies evolutionary principles from the vantage point of the evolutionary past of humans (here, the Pleistocene epoch) in order to assess present states of health and disease among populations. [10, p. 76]

Cournoyea [14, p. 478] agrees with Méthot’s distinction of Darwinian medicine and evolutionary medicine but claims that the association of the research traditions with the respective types of explanation is misleading because both research traditions use backward-looking and forward-looking reasoning. Although it is true that practitioners of evolutionary medicine sometimes “look back” (e.g., in phylogenetic analyses), they do not typically use backward-looking reasoning of the type described by Méthot because they do not apply the theoretical framework of Darwinian medicine. Nevertheless, Cournoyea’s account of evolutionary explanations of health and disease complements Méthot’s analysis. According to Cournoyea [15, p. 38], practitioners of Darwinian medicine produce coarse-grained explanations, whereas researchers in evolutionary medicine generate fine-grained explanations in time and scale. He sees the main difference between Darwinian medicine and evolutionary medicine in the time scale, and argues that Darwinian medicine is concerned with the macro domain (long-term human evolution) while evolutionary medicine is interested in the micro domain (short-term microorganism or human evolution) [14, pp. 477–478]. Cournoyea’s and Méthot’s accounts are not mutually exclusive because they emphasize different aspects. Méthot focusses on the theoretical assumptions of Darwinian medicine that underlie backward-looking explanations and Cournoyea focusses on the time scales that explanations in Darwinian medicine and evolutionary medicine encompass.

In the literature, the terms ‘evolutionary medicine’ and ‘Darwinian medicine’ are sometimes used interchangeably, and in some cases ‘evolutionary medicine’ is used as an umbrella term that includes both research traditions. However, I agree with the distinctions discussed by Méthot and Cournoyea and add a new aspect to the discussion with my analysis of explanatory and interdisciplinary integration.

## Interdisciplinary and explanatory integration

Integration is seen as one of the central characteristics of interdisciplinary research [16–20], but interdisciplinarity is also characterized by activities like interacting, blending, and linking [17, 21]. Julie Thompson Klein [17] shows that integration comes in degrees. While multidisciplinarity (understood as a juxtaposition of disciplines) can foster partial integration, interdisciplinary activities are associated with a higher degree of integration. In multidisciplinary research, “disciplines remain separate, retain their original identity, and are not questioned” [21, p. 23]. Many accounts of interdisciplinary integration emphasize the need to attain a certain degree of coherence, strive for a common goal and create or discover common ground [19, 22, 23]. According to Alan Love [23, p. 875m, emphasis omitted], interdisciplinary research is “coordinated around sets of problems (problem agendas)”. Allen Repko and Rick Szostak [19, p. 225] argue that common ground can be established through a “common ground integrator”, a concept, assumption, or theory by which insights from different fields or disciplines can be integrated.

An important question concerns the level at which integration takes place. Science studies scholars have shown that two fields can be bridged at the level of theory (e.g., via interfield theories, theory reduction, concepts [24–26]), or they can be integrated on the level of practices [27]. One can further distinguish between methodological integration, data integration and explanatory integration [28, 29]. Since proponents of Darwinian medicine want to achieve interdisciplinary integration through explanatory integration, I concentrate on the latter. Here, I follow Ingo Brigandt’s [28, pp. 296–297] characterization of explanatory integration as “the integration of ideas and explanations from different disciplines so as to yield an overall explanation of a complex phenomenon”. This account of explanatory integration captures both the notion of interdisciplinarity as involving a certain degree of unification as well as the goal of generating more complete explanations.

Proponents of Darwinian medicine frequently express the need and desire to achieve interdisciplinary integration of medicine and (evolutionary) biology, and argue that Darwinian medicine provides the conceptual framework for this “synthesis” [30, p. 1993]. Robert Perlman [31, p. 10], for example, claims: “Only recently have physicians and nonmedical biologists begun to realize that there is much to be gained by integrating these disciplines. Evolutionary medicine is based on the recognition that these different perspectives are not mutually exclusive but complementary, and that integrating them will give a richer understanding of health and disease.” According to Randolph Nesse et al. [32, p. 1080], “knowledge about evolution provides physicians with an integrative framework that links otherwise disparate bits of knowledge”. Catriona MacCallum [33, p. 680] argues that “the time has clearly come for medicine to explicitly integrate evolutionary biology into its theoretical and practical underpinnings” [see also 34, p. xiii; 7, p. 249; 4, p. 17]. As these excerpts suggest, most advocates of Darwinian medicine seem to understand the synthesis of evolutionary biology and medicine as an integration of evolutionary biology into medicine [see also 2, 35, 36]. They argue that the integration of knowledge from evolutionary biology improves our understanding of health and disease and produces more complete explanatory accounts of human diseases. Some scientists, however, emphasize that the synthesis of evolutionary biology and medicine is a “two-way street” because knowledge from evolutionary biology can inform medical practice and examples or datasets from medicine are useful resources for biologists [30, p. 1992].

In the context of Darwinian medicine, integration of evolutionary biology and medicine is usually understood as integration on the level of explanation or integration of evolutionary biology into medical education. There are many publications that address the question of how knowledge from evolutionary biology can be integrated into medical school curricula [e.g., 3, 32, 37, 38] and some authors also present suggestions of how medical examples can be used in undergraduate biology education to teach students basic principles of evolutionary biology such as genetic variation and common descent [30, p. 1995]. In this paper, however, I focus on explanatory integration and its relationship with interdisciplinary integration.

Most proponents of Darwinian medicine understand interdisciplinary integration as a synthesis of ultimate and proximate explanations of human diseases [2, 4, 39]. In this scenario, evolutionary biology provides ultimate explanations and medicine provides proximate explanations of disease. The view that human diseases have proximate and ultimate causes is one of the central theoretical assumptions of Darwinian medicine [2, pp. 6–11; 7; 14, p. 477; 30; 34, ch. 11]. Advocates of Darwinian medicine believe that the integration of an evolutionary perspective provides a more complete understanding of health and disease [36, p. 61; 37; 38, p. 576; 40].

I have already mentioned that evolutionary medicine is a less cohesive research tradition than Darwinian medicine and that scientists are not necessarily aware that their research belongs to the research tradition of evolutionary medicine. For this reason, there is no unified theoretical framework that addresses the question of how explanatory or interdisciplinary integration can or should be achieved. However, since practitioners of evolutionary medicine apply methods, practices, and concepts from evolutionary biology to address medical problems, it is worthwhile investigating whether and how evolutionary medicine generates integrated explanations and how explanatory integration relates to interdisciplinary integration within this research tradition.

In the following sections, I analyse Darwinian medicine and evolutionary medicine with respect to explanatory and interdisciplinary integration. In the next section, I take a closer look at the structure of explanations in Darwinian medicine and address the questions of whether research under the framework of Darwinian medicine produces integrated proximate-and-ultimate explanations and whether unification or blending of evolutionary biology and medicine is achieved by means of explanatory integration. I further discuss whether Darwinian medicine helps to establish common ground between researchers in evolutionary biology and medicine and fosters interdisciplinary research that is coordinated around a set of problems. In the following section, I discuss interdisciplinarity in evolutionary medicine and address the question of whether and how research in evolutionary medicine generates integrated proximate-evolutionary explanations.

## Explanatory and interdisciplinary integration in Darwinian medicine

Since advocates of Darwinian medicine aim at achieving integration on the level of explanation, I use the so-called old-friends hypothesis as an example that illustrates the structure and roles that explanations generated under the framework of Darwinian medicine play in medical and biological research. I will show that Darwinian medicine does not generate truly integrated ultimate-and-proximate explanations, but proximate explanations complemented with evolutionary concepts. I further argue that Darwinian medicine does not facilitate strong interdisciplinary integration in the sense of creating unity or blending but works as an interfield that enables cross-disciplinary exchange.

### The “old friends hypothesis”

One of the main supporters of the “old-friends hypothesis” is Graham Rook. The old-friends hypothesis is an attempt to reformulate the hygiene hypothesis “to bring it in line with Darwinian Medicine, and with the latest epidemiological and experimental evidence” [41, p. 5]. The hygiene hypothesis suggests that reduced early exposure to microbes and other organisms like helminths (parasitic worms), which play an important role in immune regulation, has led to an increase in chronic inflammatory disorders [42, 43]. Rook [44, p. 74] adds an evolutionary perspective to the discussion and argues that helminths and other organisms “have been present, inevitably and continuously, from relatively early in the evolution of the immune system” and came to play an important role in immune regulation through a process of host-parasite coevolution. He claims that some species have evolved into “friends” as a result of their long-term association with humans [45]. Helminths have evolved evasion strategies that dampen rather than disable the human immune system to ensure long-term survival within the host [46]. According to Rook [41, p. 7], the human immune system has not been able to get rid of helminths and thus has evolved to tolerate them by downregulating its immune response to avoid inappropriately strong responses that can ultimately cause harm to the host [see also 31, p. 135]. As a result of this balance between host and parasite, mild helminth infections are often asymptomatic. Rook [44, p. 79], however, takes the argument one step further and claims: “If we are thinking in a Darwinian way, we should be starting from the hypothesis that any organism that has been consistently present for a significant part of mammalian evolution might have been ‘written into’ the mammalian genome, because ‘Evolution turns the inevitable into a necessity’”. He argues that the proper development and functioning of the human immune system depends at least partially on the presence of these organisms, and that the deprivation of certain organisms such as helminths contributes to the development of chronic inflammatory diseases (e.g., autoimmune diseases) [41, 44; see also 31, p. 135].

With the old-friends hypothesis, Rook [45] proposes a possible answer to the question of why chronic inflammatory diseases (e.g., Type 1 diabetes, multiple sclerosis) are prevalent and increasing in countries of the Global North. His answer contains a proximate explanation and reference to evolutionary theory and concepts. The proximate explanation that he provides is a functional explanation. In this context, I understand functional explanations as explanations that refer to the causal role that a component of a system plays in maintaining the system’s capacity.^1^ The capacity of the immune system is to protect the organism from harmful substances from the environment, disease-causing cell changes in the body (e.g., cancer cells), and pathogens. According to Rook, the absence of helminths with their immunoregulatory capacities can lead to malfunctioning of the immune system that causes chronic inflammatory diseases. Thus, from a human perspective, the function of helminths would be to regulate the human immune system in ways that prevents it from becoming overreactive and, in the case of autoimmune diseases, attack the body’s own tissues.^2^ In his attempt to integrate this proximate explanation with an ultimate explanation, he answers the question of why helminths have immunoregulatory capacities that contribute to the proper functioning of the human immune system by referring to processes of host-parasite coevolution and a mismatch hypothesis. His claim that humans are adapted to the Pleistocene environment with constant exposure to helminths and maladapted to modern industrialized environments without helminth exposure is a paradigmatic example for a backward-looking explanation as described by Méthot [10]. Rook’s [44] entire evolutionary argument is based on this mismatch hypothesis with the Pleistocene as a reference point. To frame his argument, he uses the concept of ‘environment of evolutionary adaptedness’ (EEA) that was first introduced by psychologist John Bowlby [48] and further promoted by George Williams and Randolph Nesse in the context of Darwinian medicine. The EEA is characterized as “the ancestral environment to which a species is adapted. It is the set of selection pressures that shaped an adaptation” [49].^3^ In Darwinian medicine, the Pleistocene savannah is identified as the human EEA [4, p. 13]. The evolutionary part of the old friends hypothesis is also a course-grained evolutionary explanation in time and scale as it references only two time points that encompass a very long time-span (the Pleistocene and the present).

To support his hypothesis, Rook [44, p. 11] references studies that associate deworming of children with increased allergen sensitization, studies of animal models that suggest that helminths can oppose allergic manifestations [45, pp. 7–8], and a study of multiple sclerosis patients that showed an association of parasite infection with inhibited disease process [45, p. 8]. The parts of Rook’s [41, 44, 45] articles that deal with the evolutionary aspect relies heavily on theory (EEA, mismatch hypothesis) and contains a few references to studies that suggest the presence of helminths and other organisms in humans in the Pleistocene. Rook does not specify the evolutionary mechanisms that allegedly have led to the currently observable mechanisms of host-parasite interaction, nor does he explore other hypotheses that would explain the effects that helminths and other organisms have on our immune system.^4^ He also fails to address human or helminth evolution before or after the Pleistocene era and he doesn’t discuss reasons why up to 135,000 people die of soil transmitted helminth infection every year [52] and many more people suffer from symptoms of helminth infection (e.g., diarrhoea, abdominal pain, malnutrition, weakness, impaired growth and physical development) [53]. Another question that Rook fails to address is whether and how chronic inflammatory disorders or helminth deprivation actually reduce fitness or if the old friends hypothesis is mainly concerned with health outcomes (see Morris (2019)^5^, unpublished manuscript).^6^ Nevertheless, other scientists seem to accept the old-friends hypothesis as a plausible explanation of the increase of chronic inflammatory diseases. Taylor Smallwood and collaborators [54], for example, claim that the “hypothesis has a sound rationale given that infectious agents, including helminths, are known to be potent modulators of T cell function and that dysregulation of T cell subsets (T1 and T17) are fundamental in autoimmune disease processes including MS”. Alireza Bolourian and Zahra Mojtahedi [52] even argue that “the ‘old friend[s]’ hypothesis has been proved by studies in which the association of mucosal microbiomes with certain diseases was demonstrated”.

Studies of animal models and clinical trials suggest that helminths or helminth-derived drugs can prevent autoimmune diseases like multiple sclerosis or decrease disease severity.^7^ It is usually overlooked, however, that this evidence does not directly support the evolutionary part of the hypothesis. These studies give us information on the role of helminths in autoimmune diseases and their immunomodulating capacities, but they do not provide further evidence for the hypothesis that helminths have been present in humans in the Pleistocene or that their immunomodulating capacities have evolved in coevolution with humans and, in turn, that human immune mechanisms have evolved in coevolution with helminths in the Pleistocene era. Interestingly, although the question of why helminth immune regulation has evolved is not directly relevant for preventing or treating autoimmune diseases, several scientists mention evolutionary processes in their articles on helminth-based therapy and helminth-human interactions. Particularly in review, perspective, opinion and editorial articles, an evolutionary framework as provided by the old-friends hypothesis is used to tie findings from different fields of (bio)medical research together [e.g., 54–58]. The question that I am concerned with, however, is whether Darwinian medicine also has the potential to integrate medicine and evolutionary biology.

### The integrative potential of Darwinian medicine

The old-friends hypothesis is an exemplary case of evolutionary reasoning within the framework of Darwinian medicine. As I have argued, explanations of this kind are functional explanations of health or disease that also reference evolution. The case of the old-friends hypothesis shows that the functional explanation of the prevalence and increase of chronic inflammatory disorders in countries of the Global North is already a full-fledged explanation even without reference to evolution. The answer to the question why chronic inflammatory diseases are prevalent in countries of the Global North does not necessarily require reference to evolutionary processes [see 62 for an example]. In a medical context, a proximate explanation that shows why the presence of helminths and other organisms can prevent the human immune system from becoming overreactive would be sufficient to answer the question. This explanation could include references to immunoregulatory mechanisms, mechanisms of immune reactions, clinical trials, or studies of animal models, for example. However, reference to unspecified coevolutionary processes does not considerably (if at all) increase the explanatory power of the overall explanation and the evolutionary mismatch hypothesis alone is not a proper ultimate explanation (Morris (2019), unpublished manuscript). Thus, the old-friends hypothesis is not an explanation that integrates a proximate with a proper ultimate explanation, but rather a proximate explanation that references evolutionary concepts. This example shows that the theoretical framework of Darwinian medicine does not yield an overall explanation of a health-related problem because the evolutionary part of the explanation is not properly developed and remains speculative. Furthermore, the clinical relevance of the evolutionary part of the explanation is questionable [14, 15, 39, 63].

In the previous section, I have already mentioned that there could be another reason why Rook and other researchers include evolutionary reasoning, namely, to have a framing for their argument that ties pieces of knowledge from several fields of medical research together. This role of evolutionary theory is probably the reason why evolution is often referenced but not properly discussed in articles that summarize results from different (bio)medical studies. Another heuristic role of the old-friends hypothesis is the promotion of a new perspective on host-parasite interaction, particularly human-helminth interaction. While most research programs conceptualize helminths as pathogens that harm the host, the old-friends hypothesis emphasizes the health benefits that might arise from interactions of the human immune system with helminths or helminth-derived substances. Conceptualizing helminths as friends instead of enemies could spur new research questions that might eventually lead to the development of new drugs against chronic inflammatory disorders. This suggests that hypotheses generated under the framework of Darwinian medicine have heuristic value for medical research, as they have the potential to open up new research avenues, promote new treatments of diseases or help identifying targets for clinical intervention [see 12, 27]. To be sure, the old-friends hypothesis is certainly not the only driver of the reconceptualization of (helminth) parasites, but contributes to conceptual change.^8^ However, not only its impact on scientific research should be taken into account. Rook’s hypothesis is easy to understand, has a strong intuitive appeal and is used by several organizations (e.g., Biome Restoration, Symmbio, Wormswell, Worm Therapy) to promote DIY helminth therapy. On these websites, people can purchase helminths “for the prevention and treatment of autoimmune, allergic and inflammatory conditions” [64]. Many people do not consult a physician prior to or after the inoculation with helminths and thus create “a shadow network of patients […] trying to treat their own debilitating diseases” [65]. Thus, the old-friends hypothesis not only serves scientific but also economic interests and promotes potentially harmful self-treatment with helminths. This shows that conceptual change promoted by explanations in Darwinian medicine is not necessarily (only) positive or fruitful but can also have deleterious consequences.

Explanations like the old-friends hypothesis do not require collaboration or communication with evolutionary biologists or expert knowledge of evolutionary theory and methods because they rely on a few evolutionary concepts rather than empirical evidence, data, or complex theorizing. Knowledge of the theoretical framework of Darwinian medicine is sufficient to be able to construct a functional explanation with reference to evolution.^9^ Thus, explanations in Darwinian medicine do not stimulate collaborative interdisciplinary research that revolves around a problem agenda. The old-friends hypothesis is not a truly integrated proximate-and-ultimate explanation but an incorporation of concepts of evolutionary biology and other evolutionary concepts^10^ into a proximate explanation. As Williams and Nesse [4, p. 17, emphasis added] note, Darwinian medicine provides an “*addition* of an evolutionary perspective” to medical research. Antolin et al. [30, p. 2004, emphasis added] express a similar view in their suggestion that “*borrowing* knowledge from germ line evolution” could produce better diagnostic and prognostic strategies for cancer treatment. Rather than creating a synthesis of ultimate and proximate explanations, Darwinian medicine incorporates pieces of evolutionary theory into medicine. The potential of Darwinian medicine to integrate evolutionary biology and medicine in any strong sense is rather low because it does not promote a blending or unification of disciplines. In fact, it fosters only partial integration of evolutionary biology into medicine on a conceptual level, but there is no mutual integration of methods, practices, or data and the two disciplines remain more or less separate. One of the main reasons why explanations like the old-friends hypothesis do not foster strong interdisciplinary collaboration is the somewhat atemporal or static account of evolution provided by the framework of Darwinian medicine that does not include any details of evolutionary mechanisms or processes. In fact, mismatch hypotheses are accounts of changing environments, not of evolving species. These explanations ignore ongoing evolutionary processes and focus solely on the current state that presumably arose as an adaptation to the environment of the Pleistocene savannah.

At best, Darwinian medicine enables what Margaret Boden [20, p. 5] calls “contextualizing interdisciplinarity”, that is, “an enterprise in which one takes some account of other disciplines in teaching and/or setting one’s research-goals, but without active co-operation with those disciplines”. While the old-friends hypothesis potentially contributes to new perspectives on host-parasite interaction, new clinical insights, and therapeutic approaches in medical disciplines, it does not lead to new insights in evolutionary biology. The concept of “unilateral problem-feeding” (i.e., problem feeding without solution feeding) introduced by Henrik Thorén and Johannes Persson [67, p. 346] accounts for this asymmetrical relationship between evolutionary biology and medicine. In cases of unilateral problem-feeding, “one field (or discipline) may rely on another as a source of problems” but collaboration is not required [67, p. 346]. Hasok Chang [68, p. 280] uses the term “co-optation” to describe cases in which “one system [of practice] can be helped in its development by the use of ideas and results taken from another”. He argues that co-optation even works for incommensurable systems and does not require any interdisciplinary communication [68, p. 281]. However, there is a danger of unilateral problem-feeding or co-optation that lies in the lack of exchange and communication between researchers of the two disciplines. If researchers co-opt ideas, concepts, or results from another discipline, it is possible that they misunderstand, misinterpret, misuse, or distort them because they lack the background knowledge to properly apply or understand them; and without exchange and communication with researchers of the source discipline, the misunderstanding might remain unnoticed. This is what seems to have happened when medical researchers have incorporated evolutionary mismatch reasoning into the framework of Darwinian medicine. Rather than treating mismatch reasoning as the heuristic that it is, it is misused and misunderstood as a proper proximate explanation (Morris (2019), unpublished manuscript).

Instead of promoting integrated accounts, proponents of Darwinian medicine have created a new research tradition separate from evolutionary biology [63, p. 2]. My analysis of the old-friends hypothesis suggests that Darwinian medicine is, at least to some extent, integrated into medical theorizing, probably mainly because of its heuristic value. Cournoyea [14, p. 483] claims that Darwinian medicine generates explanations that “offer little more than speculative hypotheses about our ancestral past”. Indeed, no considerable effort has been made to find more empirical support for the evolutionary aspect of the old-friends hypothesis. To demonstrate a case of mismatch, it must be shown that a downregulation of the immune system as a reaction to persistent helminth infection was neutral or adaptive in the Pleistocene savannah and that the absence of helminths in industrialized environments causes a relatively dysfunctional immune system (see Lloyd et al. (2011)^11^, unpublished manuscript). To further support the mismatch hypothesis, it should also be shown that chronic inflammatory disorders have fitness effects in addition to health-related restrictions (see Morris (2019), unpublished manuscript). As I have mentioned in the previous section, some evidence for immune modulation through helminths and potential benefits of helminth-based therapy already exist. However, to further support the old-friends hypothesis, one needs to find evidence that the immunomodulatory properties of human pathogen helminths have indeed evolved in coevolution with humans (and not with other mammals, for example). It would also support the hypothesis to show that helminths have already been able to modulate the human immune system in the Pleistocene. Given the difficulty of obtaining knowledge about past environments, past humans, past helminths, and their interactions (e.g., by studying empirical evidence like mummies or fossils and inferring from traces as in the case of phylogenetic analysis), however, it seems that the costs of finding support for the hypothesis outweighs the benefits, at least from the perspective of a medical researcher or practitioner.

Proper backward-looking reasoning can be compared to detective work where the investigator relies on clues to reconstruct past events or processes [see 69, p. 490; 70, p. 276]. If medical researchers decided to pursue the goal of finding support for macro-evolutionary explanations of disease, it would certainly be useful to collaborate with researchers that are familiar with this kind of work, associated practices and theories (e.g., evolutionary biologists, but also researchers in paleoparasitology, palaeopathology and anthropology). At least it would be helpful to integrate data and results from these disciplines. While conceptual incorporation is sufficient to come up with plausible hypotheses, it requires stronger interdisciplinary interaction (e.g., collaboration in joint research projects, data integration) to support the hypotheses with empirical evidence. Strong collaborative interdisciplinarity would require working to achieve a common goal or creating or finding common ground between evolutionary biologists and medical researchers. Jaqueline Anderson and Florian Horn [63, p. 2], for example, suggest “the concept of a comprehensive view of the evolving healthy human” as common ground integrator.

However, maybe strong interdisciplinary integration of evolutionary biology and medicine may not be necessary to foster a fruitful exchange between the two disciplines. Maria Kronfeldner [71] identifies an implicit epistemic bias towards unity in discussions of the extension of the Modern Synthesis. The same implicit bias might also exist with respect to the connection between evolutionary biology and medicine.^12^ Even if the division of labour between evolutionary biologists and medical researchers and practitioners were maintained, theories, hypotheses, or results from one discipline could be taken up by the other discipline and generate new ways of thinking about problems or new hypotheses. This approach does not aim at unity or blending of disciplines but is based on separation [71]. Kronfeldner [71, p. 118] refers to this kind of relation between disciplines as “heuristic synthesis”. In the case of bringing together evolutionary biology and medicine under the framework of Darwinian medicine, evolutionary theory acts as an “interfield theory” [24; see 71, p. 118] that ontologically connects the two disciplines without uniting them.

This type of cross-disciplinary exchange does not require a common goal or collaborative research but might eventually foster “piecemeal integration” [72, p. 207] of evolutionary biology and medicine. Structures to support the exchange between evolutionary biologists and medical researchers have already emerged from Darwinian medicine. The International Society for Evolution, Medicine & Public Health (ISEMPH)^13^, for example, organizes annual multidisciplinary meetings and co-hosts a regularly held lecture series with the mission “to foster communication among scientists, students, clinicians and public health professionals to use evolutionary insights to improve medical research and practice, and to use studies of health and disease to advance evolutionary biology” [74]. Thus, although Darwinian medicine does not enable strong integration of evolutionary biology and medicine through integration of ultimate and proximate explanations, it works as an interfield that loosely connects the two disciplines via evolutionary theory and potentially enables heuristic synthesis [see 71].

## Integrated explanations in evolutionary medicine

The finding that Darwinian medicine does not yield truly integrated proximate-and-ultimate explanations does not imply that it is impossible to produce integrated explanations of health or disease. In this section, I discuss research in evolutionary medicine that generates integrated explanations. These explanations, however, are not produced by explanatory integration, meaning that they don’t result from synthesizing explanations from different disciplines. Instead, they are a result of interdisciplinary research at the intersection of evolutionary biology and medicine that is designed to generate entangled proximate-evolutionary explanations.

### Experimental evolution of antibiotic resistance

Since many pathogens procreate and evolve rather fast, it is relatively easy to study their evolution. While studies of human evolution usually require some kind of backward-looking approach, microbial evolution can be studied in real-time, for example by means of experimental evolution [see 10, 75]. There are several interdisciplinary research activities in fields like virology and bacteriology that are situated at the intersection of evolutionary biology and medicine. Researchers who study antibiotic resistance, the evolution of virulence, or the dynamics of infectious diseases (e.g., influenza virus infection), for example, often operate in interdisciplinary settings.^14^

Here, I present an example of an experimental evolution study of antibiotic resistance that illustrates how research in evolutionary medicine generates entangled proximate-evolutionary explanations. In experimental evolution studies, populations are studied across multiple generations to investigate “evolutionary changes occurring in experimental populations as a consequence of conditions (environmental, demographic, genetic, social, and so forth) imposed by the experimenter” [75, p. 547]. They often involve the creation of a series of evolutionary lines that are exposed to a novel environment. Tomoya Maeda and collaborators [76] conducted a laboratory evolution experiment with *Escherichia coli* (*E. coli*) bacteria to study the evolution of antibiotic resistance. Samples from a starting population of *E. coli* were used to start experimental lines that were exposed to 95 different stressors (antibiotics and non-antibiotic toxic chemicals) to analyse whether and how they evolved resistance. The researchers created one line for each of the 95 stressors plus a control without any stressor times six replicates (567 lines in total). They let the populations evolve over a period of 27 days which corresponds to approximately 250–280 generations. The researchers then produced resistance profiles for 192 of the evolved strains to study how common cross-resistance and collateral sensitivity occurred, meaning that they wanted to find out how many of the strains that evolved under a certain kind of stress also exhibit increased resistance or susceptibility to another kind of stress. They also performed genome sequencing analysis of the evolved strains to examine the genetic changes (mutations). After performing transcriptome analysis, the scientists were able to identify modular classes of gene expression profiles and analyse the relationships between genome, transcriptome and resistance profiles.

As a result of their analyses, the scientists were able to identify molecular mechanisms associated with resistance acquisition and find a connection between changes in gene expression and stress resistance. They argue that their results suggest the existence of evolutionary constraints on accessible phenotypes, meaning that “*E. coli’s* evolutionary dynamics is attributable to a relatively small number of intracellular states, indicating that it is likely equipped with only a limited number of strategies for antibiotic resistance” [78]. The researchers argue that their “results include valuable information on evolutionary constraints for antibiotic resistance, and thus, provide important insights for alternative clinical strategies” [76]. Thus, unlike explanations in Darwinian medicine, studies like the one by Maeda and collaborators [76] have clinical relevance as they point to possible strategies to prevent the emergence of antibiotic resistance.

Studies like these have a long tradition that predate the emergence of Darwinian medicine [10, p. 77]. They generate micro-domain evolutionary explanations, rely mostly on forward-looking explanations^15^ and integrate theory, methods and practices from evolutionary biology and medicine without reference to Darwinian medicine’s theoretical framework^16^ [10, pp. 77, 84; 14, p. 477]. Thus, these studies belong to the research tradition of evolutionary medicine. It seems that research activities in evolutionary medicine generate entangled proximate-evolutionary explanations more naturally than research under the framework of Darwinian medicine.

### Integrated explanations

The example shows that there are studies in biomedical research that do not only produce proximate explanations but entangled proximate-evolutionary explanations. Maeda and collaborators [76] conducted the laboratory evolution experiment because they were not only interested in the mechanisms that realize antibiotic resistance but also in the evolutionary history of these mechanisms. They wanted to know which mechanisms that realize resistance evolve in certain environments and whether the strains that evolve resistance in a certain environment are also resistant or susceptible to other kinds of stress. This research question calls for a study that addresses both evolutionary and mechanistic aspects of antibiotic resistance. Thus, from the very beginning, the study was set up to produce an explanation that references both evolutionary history and mechanisms that realize a certain biological function (resistance). Also, to design, perform and analyse the experiment, the researchers needed knowledge of concepts, methods, and practices from evolutionary biology and medicine. Thus, in this case, the integration of biology and medicine happened before the experiment was performed and the explanation was generated.

There is an important difference between forward-looking explanations in evolutionary medicine and backward-looking explanations in Darwinian medicine, namely that backward-looking explanations rely on speculative hypotheses about evolution, whereas in forward-looking explanations, the evolutionary pathways are known. Proponents of Darwinian medicine hypothesize that certain traits have evolved as adaptations to the Pleistocene environment. In the experimental evolution study by Maeda and collaborators [76], however, the state of the ancestral population and of the evolved strains is studied by means of genome and transcriptome analysis. Since the researchers have exposed the experimental *E. coli* populations to novel environments in a controlled setting, they also know which genetic changes have evolved in which environment. The difference between the two kinds of explanation is related to the time span that the respective explanations encompass. Backward-looking explanations encompass very large time spans and include events of the distant past while forward-looking explanations encompass relatively short time spans. It is much easier to generate integrated proximate-evolutionary explanations when the evolutionary pathways are known (e.g., in the case of laboratory evolution experiments) than to generate integrated explanations of long-term evolution. As I have shown in the previous section, evolutionary events of the distant past can be inferred and reconstructed from traces, but this can take a lot of time and effort and requires expertise in relevant areas of research.

Forward-looking explanations like the one presented by Maeda and collaborators [76] answer the question which causal mechanisms (e.g., mutations, gene expression changes) are relevant for particular evolutionary steps (e.g., evolution of resistance) and how they arise (e.g., in which environments they evolve). The explanatory power of these entangled proximate-evolutionary explanations thus depends on the reference to evolutionary processes. In explanations generated under the framework of Darwinian medicine, however, reference to evolution is neither necessary nor does it increase the explanatory power. While Darwinian medicine focuses on the product of research and aims at integrating proximate and evolutionary explanations on the conceptual level, interdisciplinary research in evolutionary medicine is from the very beginning designed to generate entangled proximate-evolutionary explanations.

## Integration in Darwinian medicine and evolutionary medicine

I have shown that there is a difference between Darwinian medicine and evolutionary medicine with respect to explanatory and interdisciplinary integration. Research under the framework of Darwinian medicine does not produce truly integrated proximate-and-ultimate explanations but proximate explanations that incorporate evolutionary concepts. Explanations in Darwinian medicine can be useful to integrate results from different fields of (bio)medical research and potentially promote conceptual change in medicine, but their construction does not foster interdisciplinary integration of evolutionary biology and medicine in any strong sense. However, Darwinian medicine can work as an interfield that loosely integrates evolutionary biology and medicine by enabling exchange between medical researchers and evolutionary biologists. This cross-disciplinary exchange does not require shared goals or collaborative research projects and is based on separation rather than unity. Thus, Darwinian medicine maintains the division of labour between evolutionary biologists and medical researchers. Under this framework, the focus lies on the products of medical and biological research and partial integration of proximate and evolutionary explanations happens on the conceptual level. Research activities in evolutionary medicine, on the other hand, produce genuinely integrated explanations because they are, from the very beginning, designed to produce entangled proximate-evolutionary explanations. This kind of research usually happens at the intersection of evolutionary biology and medicine where concepts, methods and practices from evolutionary biology and medicine are already integrated. Thus, in evolutionary medicine, interdisciplinary integration precedes explanatory integration. My analysis thus adds another important distinction to the philosophical discussion of the different research traditions at the intersection of evolutionary biology and medicine.

## Footnotes

1 This understanding of biological functions does not assume the existence of a *telos* (or *teloi*) in nature. However, the attribution of functions to structures or systems involves teleological reasoning to a certain extent because it conceptualizes the structures as means to an end (*telos*). For example, if one claims that the function of the heart is to pump blood, it does not necessarily mean that ‘pumping blood’ is an end inherent in nature, but rather an “epistemological reference point” [47].

2 In some of his papers, Rook [41, 45] provides details of the immunoregulatory mechanisms of helminths and other “old friends”.

3 I don’t think that the concept of the EEA defined as an ancestral environment to which a species is adapted is inherently problematic. However, its use in the context of evolutionary psychology where the Pleistocene is identified as the human EEA promotes problematic narratives, not only in popular culture but also in the scientific realm (e.g., naturalization of sex differences, man-the-hunter narratives that are used to explain human behaviour of the present) [50, 51]. A general problem with the application of the EEA combined with evolutionary mismatch reasoning is that it ignores the evolutionary processes that happened after the Pleistocene era.

4 Another possible explanation would be that immunoregulatory capacities of helminths have evolved in coevolution with other mammals maybe even before humans existed.

5 Rick Morris’s unpublished manuscript “Be fruitful and multiply. Fitness and health in evolutionary mismatch and clinical research” is part of his dissertation from the Department of Philosophy, University of California, Davis. A preprint can be found under http://philsci-archive.pitt.edu/18777/.

6 I believe that the answer to this question varies with respect to the type of disease. While allergic disorders (e.g., asthma, hay fever) don’t affect a human’s ability to produce offspring, other autoimmune diseases can have fitness reducing effects. Type 1 diabetes, for example usually occurs early in life and is associated with sexual dysfunction [59, 60].

7 See Elliott et al. [61] for a review on this topic.

8 It is difficult to evaluate the influence of a single account on conceptual change and I am not claiming that the old-friends hypothesis is the only driver of conceptual change with respect to human-helminth interactions. Concepts like ‘holobiont’ and new insights from research on human-helminth interactions are probably more influential. Nevertheless, as I have shown in the previous section, the old-friends hypothesis was well received by the scientific community.

9 However, as Pașca and Nesse [66, p. 472] note, “without evolutionary biologist collaborators, it can be difficult to distinguish viable from nonviable hypotheses”.

10 At least evolutionary mismatch reasoning is common and has a long tradition in evolutionary biology (see Lloyd et al. (2011), unpublished manuscript). The concept of EEA (environment of evolutionary adaptiveness), however, was introduced by a psychologist and is mostly used in evolutionary psychology and Darwinian medicine, not in evolutionary biology.

11 A draft by Elisabeth Lloyd, David Sloan Wilson and Elliott Sober entitled “Evolutionary Mismatch And What To Do About It: A Basic Tutorial” can be found under this link: https://evolution-institute.org/wp-content/uploads/2015/08/Mismatch-Sept-24-2011.pdf.

12 See also Gijsbers [73] for a discussion of the ideal of integration.

13 Randolph Nesse is the founding president of the International Society for Evolution, Medicine and Public Health.

14 For examples refer to studies on the evolution of viruses and bacteria at the Biozentrum of the University of Basel, Switzerland.

15 In phylogenetic or phylogenomic approaches to pathogen evolution, researchers reconstruct past evolution rather than studying real-time evolution, i.e., they “look back” [for an example see 77]. However, these studies do not rely on the theoretical framework of Darwinian medicine, e.g., they do not apply the concept of the EEA or produce mismatch hypotheses.

16 For example, they don’t mention Darwinian medicine and do not rely on mismatch hypotheses or on concepts like the EEA.
